# Timing and duration of dog walking and dog owner’s chronotype in relation to incident depression risk among middle to older-aged female nurses

**DOI:** 10.1371/journal.pone.0296922

**Published:** 2024-01-31

**Authors:** Magdalena Żebrowska, Susanne Strohmaier, Carri Westgarth, Curtis Huttenhower, Heather A. Eliassen, Shahab Haghayegh, Tianyi Huang, Francine Laden, Jaime Hart, Bernard Rosner, Ichiro Kawachi, Jorge E. Chavarro, Olivia I. Okereke, Eva S. Schernhammer

**Affiliations:** 1 Department of Epidemiology, Center for Public Health, Medical University of Vienna, Vienna, Austria; 2 Department of Livestock and One Health, Institute of Infection, Veterinary and Ecological Sciences, University of Liverpool, Liverpool, United Kingdom; 3 Department of Biostatistics, Harvard T.H. Chan School of Public Health, Boston, MA, United States of America; 4 Channing Division of Network Medicine, Department of Medicine, Brigham and Women’s Hospital and Harvard Medical School, Boston, MA, United States of America; 5 Department of Epidemiology, Harvard T.H. Chan School of Public Health, Boston, MA, United States of America; 6 Department of Nutrition, Harvard T.H. Chan School of Public Health, Boston, MA, United States of America; 7 Department of Environmental Health, Harvard T.H. Chan School of Public Health, Boston, MA, United States of America; 8 Department of Psychiatry, Massachusetts General Hospital, Boston, MA, United States of America; University of St Andrews, UNITED KINGDOM

## Abstract

**Background:**

We examined associations between dog ownership, morning dog walking and its timing and duration, and depression risk in female nurses, exploring effect modification by chronotype. We hypothesized that dog ownership and morning walking with the dog are associated with lower odds of depression, and that the latter is particularly beneficial for evening chronotypes by helping them to synchronize their biological clock with the solar system.

**Methods:**

26,169 depression-free US women aged 53–72 from the Nurses’ Health Study 2 (NHS2) were prospectively followed from 2017–2019. We used age- and multivariable-adjusted logistic regression models to estimate odds ratios (ORs) and 95% confidence intervals (95%CIs) for depression according to dog ownership, and morning dog walking, duration, and timing.

**Results:**

Overall, there was no association between owning a dog (OR_vs_no_pets_ = 1.12, 95%CI = 0.91–1.37), morning dog walking (OR_vs_not_ = 0.87, 95%CI = 0.64–1.18), or the duration (OR_>30min vs. ≤15mins_ = 0.68, 95%CI = 0.35–1.29) or timing of morning dog walks (OR_after9am vs. before7am_ = 1.06, 95%CI = 0.54–2.05) and depression. Chronotype of dog owners appeared to modify these associations. Compared to women of the same chronotype but without pets, dog owners with evening chronotypes had a significantly increased odds of depression (OR = 1.60, 95%CI = 1.12–2.29), whereas morning chronotypes did not (OR = 0.94, 95%CI = 0.71–1.23). Further, our data suggested that evening chronotypes benefited more from walking their dog themselves in the morning (OR = 0.75, 95%CI = 0.46–1.23, P_intx_ = 0.064;) than morning chronotypes.

**Conclusions:**

Overall, dog ownership was not associated with depression risk though it was increased among evening chronotypes. Walking their dog in the morning might help evening chronotypes to lower their odds of depression, though more data are needed to confirm this finding.

## Introduction

Depression is the leading cause of ill health and disability worldwide [1]. Prior research suggests, though with mixed results [2–7], that pets may provide social and psychological support [8, 9] and positively impact mental health, especially among middle- to older-aged adults [10–16]. Inconsistencies in the association between pet ownership and depression risk have been attributed to the inability of cross-sectional studies to take into account the potential for reverse causation, i.e., individuals acquiring a pet in response to loneliness or depressive symptoms [2, 17, 18]. Pet ownership could additionally reflect other unobserved characteristics of the pet owner (such as the ability to care for one’s health, or the existence of allergies) [19, 20]. In addition, dissimilarities by type of pet (most notably cats versus dogs, with scant literature on other types of pets [3]) exist regarding owners’ socioeconomic, personal and health characteristics [18] including their underlying anxiety and depression status [21–23], and with potentially opposing effects of cats versus dogs on depression risk [24].

Despite the large number of pet owners– 85 million households in the US (68%) own a pet [25]–pet ownership has been a largely untapped area for depression prevention research, including in targeted prevention contexts. In prior analyses we observed that depressive symptoms among children who experienced abuse were lower in households with pets [26]. Here, we propose that another subgroup–dog owners with an evening chronotype [27, 28] which has been associated with higher risk of depression compared to other chronotypes [29–36]–may specifically benefit from pet ownership via exposure to morning light stimulus during walks with their dogs. Indeed, the internally controlled circadian rhythm is also influenced by periodic external factors (light/dark cycle, social life), which enable daily synchronization of the biological clock with the solar rhythm [37] and thus prevent their disruption and misalignment [36]. Based on these findings, we hypothesize that dog walking in the morning could support the synchronization of people’s internal biological clock with the solar system. This could help reduce circadian misalignment and thereby be especially beneficial for evening chronotypes, potentially lowering their risk of depression.

Using the US-based Nurses’ Health Study 2 (NHS2), a longitudinal cohort of women, we examined associations between a) dog ownership, b) morning walks with the dog, and c) the timing and duration of morning walks with the dog, and risk of incident depression. Since the times of peak intellectual and physical performance fall in the morning hours for morning chronotypes and in the evening hours for evening chronotypes [38], we may speculate, that the owner’s chronotype could influence both the timing and duration of morning walks with the dog. Therefore, secondarily, we explored whether a person`s chronotype influenced the risk of depression among those walking their dog in the morning [29–35], and whether timing [39] and duration of morning walks further modified these associations.

## Methods

The NHS2 began in 1989 when 116,429 US female registered nurses aged 25–42 years returned a mailed questionnaire containing socio-demographic, lifestyle and health related characteristics. Since the start of the study, NHS2 questionnaires were mailed every two years. The study protocol was approved by the Institutional Review Board of Brigham and Women’s Hospital and the Committee on the Use of Human Subjects in Research of Harvard T.H. Chan School of Public Health (Boston, MA, USA). Voluntary return of the questionnaires implies informed consent.

In 2017, the NHS2 participants were asked the question *"Do you currently own a pet*? *(No/Yes)"* followed by the question about pet(s) type, with dog, cat and other as possible answers (multiple responses were allowed). Based on these assessments, we defined dog owners as people who owned one or more dogs (regardless of whether they also owned other types of pets) and refer to them as any-dog(s)-owners. To examine the magnitude of the influence of other accompanying pets (among dog owners) and to isolate the actual effect of the presence of a dog, we further specified the subgroup of pet owners who own only a dog/dogs (only-dog(s)-owners).

The online version of the 2017 questionnaire contained additional questions about morning walks with one’s dog: *“Do you yourself walk that dog in the mornings*?*” (Yes/No)*; and dog walking related behaviors: *“When do you typically first take your dog for a walk in the mornings*?*” (before 6am*, *between 6-7am*, *7-8am*, *8-9am*, *9-10am*, *or after 10am)* and *“Minutes typically spent outdoors while walking dog in the mornings”(<1*, *1–5*, *6–15*, *16–30*, *31–60*, *≥61)*.

Information on self-reported physician-diagnosed depression (starting in 2003) and regular use of antidepressants (starting in 1997) was obtained biennially in the NHS2 cohort. In our study, the primary outcome of interest was occurrence of incident depression in 2019, defined in the same way as in previous studies [35, 40–42], i.e. as physician-diagnosed depression or the use of antidepressants in previously depression-free individuals. To obtain a depression-free cohort at baseline, we excluded participants who had ever had a physician-diagnosed depression or used antidepressants. In sensitivity analyses, we also required a more stringent definition of incident depression based on both self-reported physician-diagnosed depression and antidepressant use [43].

Potential depression risk factors and confounders were selected based on previous findings and literature [29, 35, 44, 45]. Information on age (years), marital status (married, not married), smoking status (never, past, current smoker), retirement status (retired, not retired), physical activity (MET-hours per week) and medical comorbidity burden (≥2 self-reported major chronic diseases out of cancer, diabetes, myocardial infarction, coronary artery bypass graft surgery or percutaneous transluminal coronary angioplasty, congestive heart failure, stroke, kidney failure, chronic obstructive pulmonary disease, Parkinson’s disease, multiple sclerosis [40, 46]) were assessed at baseline (2017). Chronotype (morning (“definitely morning” or “rather more a morning than an evening type”), evening (“definitely evening” or “rather more an evening than a morning type”), neither morning nor evening type) was assessed from 2015 online questionnaire. Body mass index (BMI, in kg/m^2^; ≤25, >25) was derived from assessment of height from 1989 and self-reported weight from 2017. Median family income (as a continuous variable, in hundred thousands of US Dollars) and Census region of residence (Northeast, Midwest, South or West) were taken from 2010 Census tract data applied to geocoded addresses from the 2013 questionnaire. Alcohol consumption (in number of drinks/day) was assessed in 2015. Total number of children (continuous), and usual number of hours of sleep (≤5, 5–7, 8–9, ≥10) were assessed in 2009. In 2005, family history of major depression (yes/no; yes, if mother, father or sibling(s) have had major clinical depression) and phobic anxiety index (Crown-Crisp Experiential Index (CCI; 0–1, 2, 3, 4–5, ≥6 [47]) were assessed. Race (White, Black, Indigenous American, Asian, Hawaiian) combined information from 1989 ancestry and the 2005 race and ethnicity questions. History of physical or sexual violence by an intimate partner (yes, no) [48–50] was assessed in the 2001 Violence Questionnaire.

**Fig 1:**
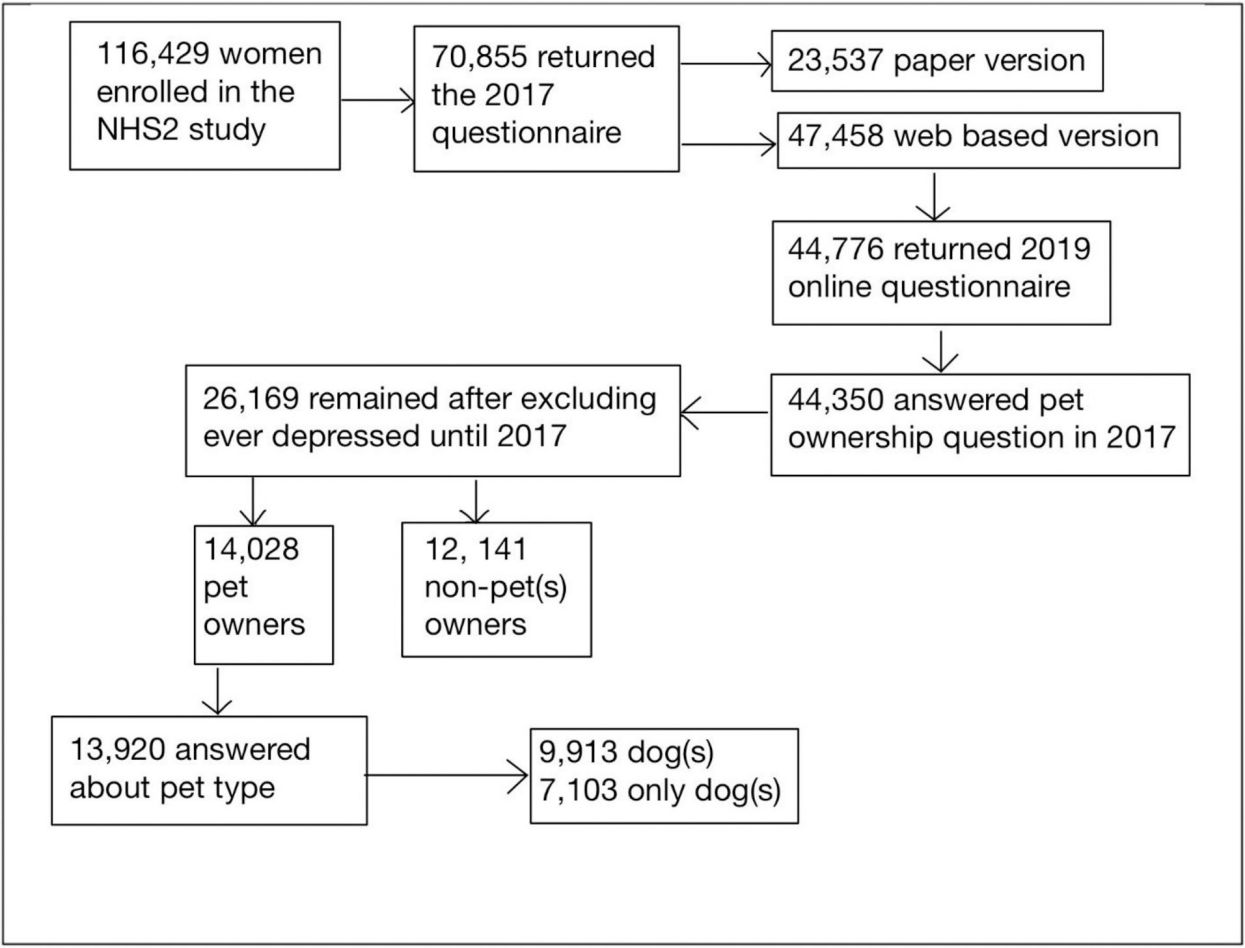
Study flow diagram illustrating the NHS2 study cohort exclusions at baseline in 2017.

Only women who had answered the online version of the NHS2 questionnaires were considered for these analyses, since primary exposures (dog walking related behaviors) were assessed only among the online participants. In 2017 online questionnaires constituted 66.8% of all returned questionnaires. Of the initial 116,429 women enrolled in NHS2, we excluded women who did not receive or return the 2017 (N = 68,971) or 2019 (N = 2,692) online questionnaire, and women who did not answer the pet ownership question in 2017 (N = 416), as well as women who had ever self-reported physician diagnosis of depression or regular use of antidepressants (N = 18,181). After these exclusions, the final study sample comprised N = 26,169 individuals (Fig 1).

### Statistical analysis

In our primary analyses, we compared each of the two defined groups of dog owners i.e. any-dog(s)-owners and only-dog(s)-owners, with the group of women who do not own pets (non-pet(s)-owners). In sensitivity analyses, we also compared each of the defined groups of dog owners to all other study participants and assessed pet ownership regardless of pet type.

When examining dog walking-related behaviors, we compared any-dog(s)-owners who declared that they themselves walked their dog in the morning to any-dog(s)-owners who did not themselves walk their dog in the morning. Because the latter group comprises a mix of dog owners who either never walk their dog themselves, or who do walk their dog but only at other times of the day, we also conducted sensitivity analyses comparing the group of women who declared morning walks with those who for certain did not take morning walks with their dogs, i.e. non-pet(s)-owners (and further with non-dog(s)-owners). To assess the possible bias caused by the presence of any-dog(s)-owners who walk their dogs at other times of the day, we also compared those who did not themselves walk their dog in the morning with non-pet(s)-owners (and non-dog(s)-owners). Regarding timing of morning walks, we defined the following categories: before 7am, 7-9am and after 9am. Since we hypothesized that earlier walks are potentially more beneficial due to earlier light stimulus, we chose before 7am as the reference group. Dog walking duration was considered in time ranges ≤15, 16–30 and ≥31 minutes with ≤15 minutes as the reference category.

We used age- and multivariable- (MV-) adjusted logistic regression to model the association between exposures and dichotomous incident depression. Multivariable models were adjusted for age, race, marital, smoking and retirement status, medical comorbidity burden, BMI, median family income, region of residence, number of children, alcohol consumption, usual number of hours of sleep, family history of depression, phobic anxiety index, intimate partner violence and chronotype. To avoid non-convergence due to low case numbers within certain exposure-defined subsamples, we applied Firth´s correction [51], which provides bias reduction as well as finite and consistent estimates even in case of separation [52].

To assess the role of chronotype as a potential effect modifier, we performed stratified analyses for morning, evening, and neither morning nor evening chronotypes. Additionally, we tested for interactions between considered exposures and chronotype. All analyses were conducted using SAS software, version 9.4 (SAS Institute, Inc., Cary, NC).

## Results

Table 1 offers baseline characteristics of the 26,169 women who formed our base population by pet ownership status. Briefly, compared to women without a pet, dog owners were more likely to be White, married, have a higher phobic anxiety index, and more frequently report physical or sexual intimate partner violence. The basic characteristics of the study participants in groups defined by the dog-walking related exposures are presented in the S1-S3 Tables in S1 File.

**Table 1:** Age and age-adjusted characteristics of participants in Nurses’ Health Study 2 who were free of depression at baseline and reported on pet ownership in 2017 stratified by pet- and dog-ownership (N = 26,169).

Characteristic*	Overall study sample N = 26,169	No pet N = 12,141	Any pet(s) N = 14,028	Any dog(s) N = 9,913	Only dog(s) N = 7,103	Missing (%)
Age (years)	63.1± 4.6	63.8± 4.4	62.4± 4.6	62.2± 4.6	62.2± 4.6	0.0
Race (%)	0.0					
White	96.5	95.4	97.4	97.3	97.1	
Black	1.4	2.2	0.7	0.8	1.0	
American indigenous	0.4	0.2	0.5	0.5	0.5	
Asian	1.6	2.1	1.2	1.2	1.3	
Hawaiian	0.1	0.1	0.1	0.2	0.2	
Region of residence (%)	1.7					
Northeast^a^	31.3	33.4	29.4	28.2	29.2	
Midwest^b^	30.0	31.3	28.8	28.4	29.3	
South^c^	19.3	18.2	20.3	21.8	21.4	
West^d^	17.6	15.7	19.5	19.5	18.0	
Married (%)	79.3	79.3	79.0	81.0	82.6	0.3
Median family income (in hundred thousands of US Dollar)	0.9± 0.3	0.9± 0.3	0.8± 0.3	0.8± 0.3	0.9± 0.3	1.7
Retired (%)	38.6	40.0	37.0	37.1	34.8	0.0
Number of children (total parity)	1.9± 1.2	2± 1.2	1.9± 1.2	2± 1.2	2± 1.2	0.0
BMI, (kg/m^2^)	26.8± 6	26.6± 6	27± 6	27± 5.9	26.9± 5.9	3.5
Alcohol consumption (drinks/day)	0.5± 0.7	0.5± 0.7	0.5± 0.7	0.5± 0.7	0.5± 0.7	0.4
Smoking status (%)	0.0					
Never/past smoker	97.6	97.9	97.4	97.5	97.5	
Current smoker	2.4	2.1	2.6	2.5	2.5	
Chronotype^e^ (%)	0.0					
Morning chronotype	60.4	61.5	59.3	59.8	60.5	
Evening chronotype	27.5	27.2	27.9	26.9	26.2	
Neither morning nor evening	4.3	4.4	4.2	4.0	3.9	
Hours of sleep (%)	2.1					
<5h	0.2	0.2	0.2	0.3	0.3	
5-7h	60.7	60.7	60.6	59.9	60.1	
8-9h	36.4	36.6	36.2	36.6	36.6	
≥10h	0.6	0.6	0.6	0.6	0.5	
Phobic anxiety index (%) (Crown Crisp CCI)	7.5					
CCI: 0–1	52.1	53.5	50.8	49.9	50.9	
CCI: 2	20.6	20.2	21.0	21.1	20.8	
CCI: 3	10.3	10.2	10.6	10.4	10.4	
CCI: 4–5	7.8	7.6	7.9	8.3	7.6	
CCI: 6 or higher	1.7	1.6	1.7	1.8	1.7	
Physical or sexual intimate partner violence (%)	21.3	18.4	24.0	24.5	22.8	21.4
Family history of major depression (%)	14.2	14.0	14.4	13.9	13.5	0.0
Medical comorbidity burden (%)	0.9	0.9	0.9	1.0	1.0	0.0
Total activity MET-hours/week	30.7± 32.9	30.6± 32.4	30.7± 33.3	31.2± 33.6	31.2± 32.9	0.0

* Values are means ± SD or percentages

^a^ Northeast: PA, NY, NJ, ME, NH, MA, RI, CT, VT

^b^ Midwest: MI, WI, IN, OH, IL, ND, NE, MN, KS, SD, IA, MO

^c^ South: MS, KY, AL, TN, SC, WA, NC, DE, MD, FL, VA, GA, WV, AR, TX, OK, LA

^d^ West: WY, ID, NM, CO, AZ, MT, UT, NV, AK, WA, OR, HI, CA

^e^ Morning: definitely a morning or more of a morning than an evening type

Evening: definitely an evening or more of an evening than a morning

Neither: neither morning nor evening type

^f^ Crown-Crisp Experiential Index (CCI), score range 0–16.

During follow-up (2017–2019), a total of 445 cases of incident depression (255 among any-pet(s)-owners, 182 among any-dog(s)-owners, 190 among non-pet(s)-owners) were accrued using our primary definition of depression, and 86 cases (58 among any-pet(s)-owners, 37 among any-dog(s)-owners, 28 among non-pet(s)-owners) using the more stringent definition.

Results from age-adjusted regression models were largely similar to those from MV-adjusted, and we therefore focus on the latter estimates. Overall, there was no statistically significant association between pet or dog ownership, and risk of incident depression. Compared to women without a pet, there was no evidence that those with a pet (OR_any-pet(s)_ = 1.11, 95%CI = 0.91–1.34) or a dog (OR_any-dog(s)_ = 1.12, 95%CI = 0.91–1.38) had higher odds of depression, and this was attenuated even further for the dog owners when excluding other pets (OR_only-dog(s)_ = 1.09, 95%CI = 0.87–1.37; Table 2). Results remained largely similar when changing the reference group to non-dog(s)-owners (S4 Table in S1 File).

**Table 2:** Association of pet ownership and incident depression^(a)^ risk of participants in Nurses’ Health Study 2 who were free of depression at baseline and reported on pet ownership. Risk estimates are odds ratios (OR) with 95% confidence intervals (CI). (N = 26,169).

			OR (95%CI)	
Exposure	N obs.	N case	Age adj.	MV adj.
No pet (ref)	12,141	190	1.00	1.00
Any pet(s)	14,028	255	1.12 (0.92, 1.35)	1.10 (0.91, 1.33)
Any dog(s)	9,913	182	1.13 (0.92, 1.39)	1.12 (0.91, 1.37)
Only dog(s)	7,103	125	1.09 (0.87, 1.37)	1.09 (0.87, 1.37)

^(a)^ Incident depression defined as a self-reported diagnosis of depression or self-reported antidepressants use

N obs.: number of observations used; N case: number of depression cases; Age adj.: age-adjusted model

MV adj.: model adjusted for age, ethnicity, marital, smoking and retirement status, medical comorbidity, BMI, median family income, region of residence, number of children, alcohol consumption, usual number of hours of sleep, family history of depression, phobic anxiety index, intimate partner’s violence and chronotype.

Among the dog owners, overall, there was no statistically significant association between morning dog walking and risk of incident depression (dog owners who did versus did not walk their dog in the morning: OR = 0.87, 95%CI = 0.64–1.18; Table 3). Comparisons with non-pet(s)-owners (S5 Table in S1 File) and with non-dog(s)-owners (S6 Table in S1 File) also revealed null results, both for dog owners who walked their dogs in the morning (versus non-pet(s)-owners: OR = 1.00, 95%CI = 0.76–1.32; versus non-dog(s)-owners: OR = 0.98, 95%CI = 0.75–1.27), as well as for those who did not walk with the dog in the morning (versus non-pet(s)-owners: OR = 1.19, 95%CI = 0.94–1.51; versus non-dog(s)-owners: OR = 1.14, 95%CI = 0.91–1.43). Further, there was no evidence that duration of walking with the dog in the morning influenced odds of depression (OR_16-30min vs. ≤15mins_ = 1.10, 95%CI = 0.63–1.93; OR_>30min vs. ≤15mins_ = 0.68, 95%CI = 0.35–1.29; Table 3). Lastly, also timing of dog walking did not alter the risk of depression materially: compared to women who walked their dog before 7am, those who walked them between 7-9am (OR = 0.94, 95%CI = 0.53–1.68) or later than 9am (OR = 1.06, 95%CI = 0.54–2.05) had comparable odds of depression (Table 3).

**Table 3:** Association of dog-walking-related behaviors among dog owners and incident depression^(a)^ risk of participants in Nurses’ Health Study 2 who were free of depression at baseline. Risk estimates are odds ratios (OR) with 95% confidence intervals (CI). (N = 9,835).

				OR (95%CI)	
Exposure		N obs.	N cases	Age adj.	MV adj.
Do you yourself walk that dog in the mornings?	No (ref.)	5,672	111	1.00	1.00
	Yes	4,163	68	0.85 (0.63,1.16)	0.87 (0.64,1.18)
Minutes typically spent outdoors while walking dog in the mornings	≤15 min. (ref.)	1,370	23	1.00	1.00
	16–30 min.	1,451	28	1.16 (0.67,2.03)	1.10 (0.63,1.93)
	>30 min.	1,345	16	0.72 (0.38,1.35)	0.68 (0.35,1.29)
When do you typically first take your dog for a walk in the mornings?	Before 7am (ref.)	1,585	23	1.00	1.00
	7-9am	1,677	27	1.13 (0.65,1.98)	0.94 (0.53, 1.68)
	After 9am	898	17	1.35 (0.71, 2.52)	1.06 (0.54, 2.05)

^(a)^ Incident depression defined as a self-reported diagnosis of depression or self-reported antidepressants use

N obs.: number of observations used; N case: number of depression cases; Age adj.: age-adjusted model; MV adj.: model adjusted for age, ethnicity, marital, smoking and retirement status, medical comorbidity, BMI, median family income, region of residence, number of children, alcohol consumption, usual number of hours of sleep, family history of depression, phobic anxiety index, intimate partner’s violence and chronotype.

In a separate set of analyses, we stratified dog owners by chronotype (Table 4). First, in line with previous research, we found that evening chronotypes had statistically significantly elevated risk of depression (OR = 1.60, 95%CI = 1.12–2.29), whereas this was not the case for morning chronotypes (OR = 0.94, 95%CI, 0.71–1.23), when compared to the respective chronotypes without a pet. Next, we examined whether dog walking differentially changed the odds of depression among evening vs. morning chronotypes. We found that, if the evening chronotypes walked their dog themselves in the morning, their odds of depression decreased (although not statistically significantly) when compared to evening chronotypes who did not walk their dog themselves in the morning (OR = 0.75, 95%CI = 0.46–1.23, P_intx_ = 0.064; Table 5). By contrast, dog walking in the morning did not alter the odds of depression among the morning chronotypes (OR = 0.94, 95%CI, 0.63–1.40, Table 5).

**Table 4:** Association of pet ownership and incident depression^(a)^ risk of participants in Nurses’ Health Study 2 who were free of depression at baseline and reported on pet ownership, stratified by chronotype^(b)^. Risk estimates are odds ratios (OR) with 95% confidence intervals (CI). (N = 26,169).

	Morning chronotype				Evening chronotype				Neither morning nor evening chronotype				p_int_ ^(c)^
			OR (95%CI)				OR (95%CI)				OR (95%CI)		
Exposure	N obs.	N case	Age adj.	MV adj.	N obs.	N case	Age adj.	MV adj.	N obs.	N case	Age adj.	MV adj.	
**No pet (ref.)**	7,589	122	1.00	1.00	3,480	53	1.00	1.00	834	14	1.00	1.00	
Any pet(s)	8,589	136	0.95(0.74,1.22)	0.94 (0.74,1.21)	4,150	98	1.53 (1.09,2.14)	1.52 (1.09,2.13)	935	17	0.93 (0.46,1.89)	0.93 (0.50,1.76)	0.078
Any dog(s)	6,145	97	0.95 (0.72,1.24)	0.94 (0.71,1.23)	2,845	71	1.62 (1.13,2.33)	1.60 (1.12,2.29)	647	12	0.95 (0.44,2.06)	0.93 (0.47,1.84)	0.074
Only dog(s)	4,470	66	0.88 (0.65,1.19)	0.88 (0.65,1.18)	1,981	49	1.65 (1.11,2.45)	1.61 (1.09,2.36)	449	9	1.09 (0.48,2.51)	0.99 (0.47,2.06)	0.104

^(a)^ Incident depression defined as a self-reported diagnosis of depression or self-reported antidepressants use

^(b)^ Morning: definitely a morning or more of a morning than an evening type; evening: definitely an evening or more of an evening than a morning; neither: neither morning nor evening type

^(c)^: p values for an interaction of a specific exposure with chronotype (morning, evening or neither morning nor evening) in the MV adj. model

N obs.: number of observations used; N case: number of depression cases; Age adj.: age-adjusted model; MV adj.: model adjusted for age, ethnicity, marital, smoking and retirement status, medical comorbidity, BMI, median family income, region of residence, number of children, alcohol consumption, usual number of hours of sleep, family history of depression, phobic anxiety index and intimate partner’s violence.

**Table 5:** Association of dog-walking-related behaviors among dog owners and incident depression^(a)^ risk of participants in Nurses’ Health Study 2 who were free of depression at baseline, stratified by chronotype^(b)^. Risk estimates are odds ratios (OR) with 95% confidence intervals (CI). (N = 9,835).

		Morning chronotype				Evening chronotype				p_int_^(c)^
				OR (95%CI)				OR (95%CI)		
Exposure		N obs.	N case	Age adj.	MV adj.	N obs.	N case	Age adj.	MV adj.	
Do you yourself walk that dog in the mornings?	No (ref.)	3,169	52	1.00	1.00	1,830	50	1.00	1.00	0.064
	Yes	2,804	41	0.93 (0.61,1.39)	0.94 (0.63,1.40)	993	21	0.77 (0.46,1.29)	0.75 (0.46,1.23)	
Minutes typically spent outdoors while walking dog in the mornings	<15min. (ref.)	877	14	1.00	1.00	372	7	1.00	1.00	0.484
	16–30 min.	976	19	1.22 (0.62, 2.47)	1.20 (0.60,2.46)	359	7	1.01 (0.36,2.89)	0.88 (0.30,2.56)	
	>30 min.	951	8	0.55 (0.22,1.26)	0.55 (0.22,1.30)	266	7	1.38 (0.48,3.94)	1.00 (0.33,3.01)	
When do you typically first take your dog for a walk in the mornings?	Before 7am (ref.)	1,214	17	1.00	1.00	247	5	1.00	1.00	0.639
	7–9 am	1,098	15	1.01 (0.50,2.03)	0.95 (0.46,1.96)	423	7	0.77 (0.25,2.49)	0.53 (0.16,1.76)	
	After 9am	496	8	1.24 (0.51,2.77)	1.16 (0.47,2.69)	321	9	1.28 (0.45,4.03)	0.77 (0.25,2.56)	

^(a)^ Incident depression defined as a self-reported diagnosis of depression or self-reported antidepressants use

^(b)^ Morning: definitely a morning or more of a morning than an evening type; evening: definitely an evening or more of an evening than a morning; neither: neither morning nor evening type; For the neither group number of cases in subgroups were not enough to perform analysis (information matrix singular)

^(c)^: p values for an interaction of a specific exposure with chronotype (morning, evening or neither morning nor evening) in the age adjusted model (for MV adj. models information matrix was singular) N obs.: number of observations used; N case: number of depression cases

Age adj.: age-adjusted model; MV adj.: model adjusted for age, ethnicity, marital, smoking and retirement status, medical comorbidity, BMI, median family income, region of residence, number of children, alcohol consumption, usual number of hours of sleep, family history of depression, phobic anxiety index and intimate partner’s violence.

Last, we examined whether chronotype modified the association between timing and duration of walking one’s dog and depression odds, and none the results achieved significance. The association of duration of walking one’s dog in the morning and depression risk did not substantially differ among dog owners with morning chronotypes (OR_>30min vs. ≤15mins_ = 0.55, 95%CI = 0.22–1.30), versus evening chronotypes (OR_>30min vs. ≤15mins_ = 1.00, 95%CI = 0.33–3.01; P_intx_ = 0.48); Table 5. For evening chronotypes, we observed some weak indication that later walks with the dog might be more beneficial in terms of depression risk (compared to walking the dog before 7am: for those who walked their dog between 7-9am, OR = 0.53, 95%CI, 0.16–1.76; after 9am, OR = 0.77, 95%CI, 0.25–2.56; P_intx_ = 0.64; Table 5). Timing of dog walking did not matter for the morning chronotypes, i.e. if they walked their dog after 7am (compared to walking the dog before 7am: for those who walked their dog between 7-9am, OR = 0.95, 95%CI, 0.46–1.96; after 9am, OR = 1.16, 95%CI, 0.47–2.69; Table 5) compared to morning chronotypes who walked their dogs before 7am.

In sensitivity analyses considering a more stringent definition of depression (i.e., both physician-diagnosed depression *and* use of antidepressants, S8-S10 Tables in S1 File), results were largely similar though power limited a detailed exploration of effect modification by chronotype in the associations between dog ownership related behavior and depression.

## Discussion

In our study, overall, neither pet nor dog ownership *per se*, or dog walking related behavior, was associated with depression risk. We examined effect modification by chronotype and found depression risk to be higher among dog owners with evening chronotypes (compared to evening chronotypes without a pet), with some indication that walking their dog in the morning might be beneficially for the evening chronotypes.

Our overall null findings are consistent with previous literature on pet and dog ownership and depression risk, suggesting mixed evidence. Pet owners differ systematically from non-pet owners [3], rendering their general comparison potentially too crude to account for all possible variations in the human-animal relationship. Additionally, methodological challenges exist with the need for more detailed, longitudinal studies to address the temporal relationship between pet ownership and incident depression.

Dog owners also should not be assumed to be a homogeneous group as the potential health benefits of dog ownership in this group are more likely due to the specific dog-related activities that owners engage in, than due to dog ownership *per se* [2]. For example, up to 40% of all dog owners [53] do not walk with their dogs at all and therefore health benefits associated with increased walking cannot be expected in this subgroup [2]. Furthermore, dog walking, which is a common form of pet related human-animal interaction (HAI), is based on the partnership between dogs and their owners [54]. Walking with a dog can substantially differ from walking without a dog [53] and, depending on the motivation of the owner, different types of dog walking behaviors can occur (functional, recreational) [55]. Likewise, dog walking-related behaviors may reflect dogs’ and owners’ specific features, including potential resilience factors such as owners’ attitude and commitment [56], self-determination [54] or attachment to pet [57], possibly mediating the relationship between dog walking and mental health outcomes. Nonetheless, dog walking behaviors have previously been related to human health and wellbeing [58–61] through increased physical activity [9, 62–65] and by providing opportunities for social interaction [9, 20, 58]. Our findings showing no association between dog walking related behaviors and depression risk are only partially in line with these earlier observations. However, we were unable to take into account the dog owner’s attachment to their pet, which might have altered our results.

A possible link between dog ownership and chronotype has not been extensively researched. Sládek et al [39] showed that dog ownership was significantly associated with late chronotype in young (18–39 years old) but not older subjects, whereas the subjectively assessed owner´s chronotype remained unaffected by the presence of a dog. Similarly, Randler et al [66] showed that dogs synchronize to humans in their sleep patterns and it is possible that they therefore may not contribute to synchronization of owner´s circadian clock with the solar system [39, 66]. Yet, walking one’s dog in the morning, even if in the subjective (late) morning of an evening chronotype, will still provide a bright light stimulus that may otherwise not have been experienced at this time of the day. As such, one could speculate that both morning and evening chronotypes should benefit from walking their dogs in the morning, especially if the duration provides a long enough exposure to bright light to impact their circadian system thereby strengthening their sleep. Alternatively, morning chronotypes might be more likely to prefer earlier and longer morning walks, while owners with an evening chronotype may tend to shift morning walks to later morning hours, or only walk their dogs in the afternoons/evenings. Thus, individuals with a morning chronotype would receive a daily morning light stimulus, which would contribute to an even better synchronization of their biological clock with the solar system, further lowering their risk of depression. In contrast, those with an evening chronotype postpone the morning light stimulus to later hours, and perhaps by participating in late night walks, they expose themselves to artificial light, which further contributes to shifting and desynchronizing their biological clock and higher depression risk. Our findings of evening chronotypes benefitting from morning walks with their dog, and with some suggestion of timing, but not duration, of morning walks playing a role in these associations, are novel and require confirmation in larger data sets.

Our study has several strengths of note. First, in contrast to cross-sectional studies, the longitudinal design of the NHS2 cohort allowed us to formulate causal hypotheses. We were able to account for a large number of potential confounding variables, though uncontrolled confounding always remains a concern. We had detailed information on morning dog walking behavior and chronotype, which is unique in prospective settings of large cohort studies. Further, we were able to isolate the effect of dog ownership and morning walks with the dog by focusing on individuals who had only dogs but no other pets.

We are also aware of some limitations of our study, which may have affected the generalizability of our results or have introduced bias. First, all the participants in our study were female middle- to older-aged nurses, predominantly of white race and the data analyzed came exclusively from electronic questionnaires. To the best of our knowledge, however, there is no other such large cohort containing information on pet ownership, health status, family medical history and chronotype. As trained health professionals, nurses provide more complete and accurate information than women from the general population, minimizing under- or misreporting of depression diagnoses and ensuring high data quality. The high homogeneity of study participants ensures minimal socioeconomic differences and increases internal accuracy. Second, all data are self-reported, which, especially in the context of depression and antidepressant use, can introduce misclassification bias. Sensitivity analyses considering more stringent definition of depression were aimed to address this bias, they were however limited in power due to very small case numbers. However, given the prospective nature of our analyses, we would expect misclassification bias to be random and therefore likely having attenuated our results. Third, differential self-selection into pet ownership may have biased our results. Indeed, this is easily observed in our baseline data, where, compared to no-pet owners, dog owners more often reported intimate partner violence and higher anxiety scores, suggesting that dog ownership may in part be an indicator of a specific familial environment or psychological risk factors. Analyses among dog owners only, e.g., when examining timing and duration of morning dog walking, should have alleviated this concern. Fourth, we expect that the geographical and seasonal variation in the light exposure is another source of bias. However, assuming that the timing and duration of dog walking for each person is approximately stable over all seasons, the overall annual exposure to the sunlight during morning walks of a fixed time and duration is approximately the same over all regions at the same latitude. In addition, existing time zones should partially reduce this latitude variation in daylight exposure. The other component of the geographic variation, namely the day length we are addressing by adjusting our models for the region of residence.

In conclusion, in this study of middle aged and older women, pet ownership, dog ownership and dog-related HAI in the form of walking in the morning were not significantly associated with incident depression risk. We identified subgroups of dog owners for whom specific dog walking practices influenced their risk of depression. Our results point to the need for a deeper consideration of the topic of HAI, potentially considering strength of human-animal attachment, and add a novel element of chronotype in our understanding of the role of dogs in improving the health and well-being of their owners. This knowledge can potentially contribute to improving preventive and therapeutic strategies in this specific group of women.

## Supporting information

S1 File (DOCX)
