# Supplementary material for: Timing and duration of dog walking and dog owner’s chronotype in relation to incident depression risk among middle to older-aged female nurses
Source: PLoS One. 2024 Jan 31;19(1):e0296922. doi: 10.1371/journal.pone.0296922 (PMC10829988; doi:10.1371/journal.pone.0296922)
Supplement: S1 File — (DOCX) [file pone.0296922.s001.docx]

**Supplementary tables for the manuscript “Timing and duration of dog walking and dog owner’s chronotype in relation to incident depression risk among middle to older-aged female nurses”.**

**List of supplementary tables:**

1. Characteristics of study participants in categories defined by dog walking related exposures

- Table S1 Age and age-adjusted characteristics of Nurses` Health Study 2 participants who owned a dog in 2017 (N=9,835), by dog walking status.
- Table S2 Age and age-adjusted characteristics of Nurses` Health Study 2 participants who owned a dog in 2017 and walked the dog in the mornings (N=4,163), by dog walking timing.
- Table S3 Age and age-adjusted characteristics of Nurses` Health Study 2 participants who owned a dog in 2017 and walked the dog in the mornings (N=4,163), by dog walking duration.

1. Results for dog ownership and dog walking related exposures with broader definition of reference groups

- Table S4 Association of pet ownership and incident depression^(a)^ risk of participants in Nurses` Health Study 2 who were free of depression at baseline and reported on pet ownership. Risk estimates are odds ratios (OR) with 95% confidence intervals (CI).(N=26,169)
- Table S5: Association of morning dog walking preferences among dog owners compared to no pet owners and incident depression^(a)^ among the participants of Nurses` Health Study 2. Risk estimates are odds ratios (OR) with 95% confidence intervals (CI).(N=21,975)
- Table S6: Association of morning dog walking preferences among dog owners compared to no-dog owners and incident depression^(a)^ among the participants of Nurses` Health Study 2. Risk estimates are odds ratios (OR) with 95% confidence intervals (CI).(N=26,088)
- Table S7: Association of dog-walking-related behaviors among dog owners and incident depression^(a)^ risk among participants of the Nurses` Health Study 2 stratified by chronotype^(b)^. Risk estimates are odds ratios (OR) with 95% confidence intervals (CI). (N=23,738).

1. Results for strict definition of depression (self-reported diagnosis of depression and self-reported antidepressants use):

- Table S8: Association of pet ownership and incident depression^(a)^ risk of participants in Nurses` Health Study 2 who were free of depression at baseline and reported on pet ownership. Risk estimates are odds ratios (OR) with 95% confidence intervals (CI). (N=26,169).
- Table S9: Association of dog-walking-related behaviors among dog owners and incident depression^(a)^ risk of participants in Nurses` Health Study 2 who were free of depression at baseline and reported on pet ownership. Risk estimates are odds ratios (OR) with 95% confidence intervals (CI).(N=21,975).

Table S10: Association of pet ownership and incident depression(^a)^ risk of participants in Nurses` Health Study 2 who were free of depression at baseline and reported on pet ownership, stratified by chronotype. Risk estimates are odds ratios (OR) with 95% confidence intervals (CI). (N=23,780).

## Characteristics of study participants in categories defined by dog walking related exposures

| **Table S1** Age and age-adjusted characteristics of Nurses` Health Study 2 participants who owned a dog in 2017 (N=9,835), by dog walking status. | | | |
| --- | --- | --- | --- |
|  | Not walking dog in mornings n=5,672 | Walking dog in mornings n=4,163 | Missing |
| Age in years^*^ | 61.8± 4.6 | 62.7± 4.6 | 0.0 |
| Race (%) |  | | 0.0 |
| White | 97.3 | 97.6 |  |
| Black | 0.9 | 0.6 |  |
| American indigenous | 0.4 | 0.6 |  |
| Asian | 1.2 | 1.1 |  |
| Hawaiian | 0.2 | 0.2 |  |
| Region of living (%) |  | | 2.4 |
| Northeast^a^ | 26.9 | 30.1 |  |
| Middlewest^b^ | 31.4 | 25.8 |  |
| South^c^ | 22.4 | 21.3 |  |
| West^d^ | 16.9 | 20.9 |  |
| Married (%) | 84.3 | 79.4 | 0.3 |
| Median family income(in hundred thousands of US Dollar) | 0.8± 0.3 | 0.9± 0.3 | 2.2 |
| Retired (%) | 29.0 | 37.0 | 0.0 |
| Number of children (total parity) | 2.1± 1.2 | 1.8± 1.2 | 0.0 |
| BMI, (kg/m2) | 27.8± 6.3 | 25.9± 5.2 | 4.3 |
| Alcohol consumption (drinks/d) | 0.4± 0.7 | 0.5± 0.8 | 0.5 |
| Smoking status (%) |  | | 0.0 |
| Never or Past smoker | 97.2 | 97.8 |  |
| Current smoker | 2.8 | 2.2 |  |
| Chronotype^e^: |  | |  |
| Morning | 55.8 | 64.0 |  |
| Evening | 29.9 | 23.5 |  |
| Neither | 4.2 | 3.6 | 0.0 |
| Hours of sleep (%) |  | | 2.7 |
| <5 | 0.3 | 0.3 |  |
| 5-7h | 60.6 | 59.1 |  |
| 8-9h | 35.7 | 37.6 |  |
| ≥10h | 0.7 | 0.5 |  |
| Phobic anxiety index (%) |  | | 9.3 |
| CCI: 0-1 | 48.3 | 52.2 |  |
| CCI: 2 | 21.9 | 19.9 |  |
| CCI: 3 | 10.4 | 10.0 |  |
| CCI: 4-5 | 8.4 | 8.0 |  |
| CCI: 6 or higher | 1.7 | 1.8 |  |
| Physical or sexual intimate partner`s violence (%) | 23.4 | 23.9 | 22.9 |
| Family history of major depression, (%) | 13.5 | 14.0 | 0.0 |
| Medical comorbidity burden, (%) | 1.1 | 0.5 | 0.0 |
| Total activity MET-hours/week | 27± 31.5 | 37.6± 36.4 | 0.0 |
| ^*^ Values are means ± SD or percentages;  ^a^ Northeast: PA, NY, NJ, ME, NH, MA, RI, CT, VT;  ^b^ Midwest: MI, WI, IN, OH, IL, ND, NE, MN, KS, SD, IA, MO;  ^c^ South: MS, KY, AL, TN, SC, WA, NC, DE, MD, FL, VA, GA, WV, AR, TX, OK, LA;  ^d^ West: WY, ID, NM, CO, AZ, MT, UT, NV, AK, WA, OR, HI, CA;  ^e^ Morning: definitely a morning or more of a morning than an evening type;  Evening: definitely an evening or more of an evening than a morning;  Neither: neither morning nor evening type;  ^f^ Crown-Crisp Experiential Index (CCI), score range 0-16. | | | |

| **Table S2** Age and age-adjusted characteristics of Nurses` Health Study 2 participants who owned a dog in 2017 and walked the dog in the mornings (N=4,163), by dog walking timing. | | | | |
| --- | --- | --- | --- | --- |
|  | When do you typically first take your dog for a walk in the mornings? | | | |
|  | Before 7am N=1,585 | Between 7-9am N=1,677 | After 9am N=898 | Missing |
| Age in years^*^ | 62± 4.5 | 63± 4.5 | 63.3± 4.7 | 0.0 |
| Race (%) |  | | | 0.0 |
| White | 96.7 | 98.0 | 97.2 |  |
| Black | 0.8 | 0.4 | 0.8 |  |
| American indigenous | 0.6 | 0.4 | 0.9 |  |
| Asian | 1.3 | 1.0 | 1.1 |  |
| Hawaiian | 0.1 | 0.2 | 0.2 |  |
| Region of living (%) |  | | | 2.2 |
| Northeast^a^ | 32.8 | 28.4 | 25.4 |  |
| Middlewest^b^ | 22.5 | 24.4 | 31.2 |  |
| South^c^ | 22.1 | 24.0 | 15.6 |  |
| West^d^ | 20.4 | 21.3 | 25.7 |  |
| Married (%) | 73.4 | 81.2 | 83.6 | 0.2 |
| Median family income(in hundred thousands of US Dollar) | 0.9± 0.3 | 0.9± 0.4 | 0.9± 0.3 | 2.0 |
| Retired (%) | 29.0 | 46.1 | 50.0 | 0.0 |
| Number of children (total parity) | 1.8± 1.2 | 1.8± 1.2 | 1.9± 1.1 | 0.0 |
| BMI, (kg/m^2^) | 26.3± 5.5 | 25.7± 5.1 | 25.7± 4.9 | 3.9 |
| Alcohol consumption (drinks/d) | 0.5± 0.7 | 0.5± 0.8 | 0.6± 0.8 | 0.4 |
| Smoking status (%) |  | | | 0.0 |
| Never or Past smoker | 97.8 | 97.6 | 98.1 |  |
| Current smoker | 2.2 | 2.4 | 1.9 |  |
| Chronotype^e^ (%) |  | | | 0.0 |
| Morning | 71.9 | 62.6 | 53.8 |  |
| Evening | 15.4 | 24.9 | 33.3 |  |
| Neither | 3.1 | 3.9 | 4.3 |  |
| Hours of sleep (%) |  | | | 2.7 |
| <5h | 0.6 | 0.0 | 0.1 |  |
| 5-7h | 62.4 | 59.6 | 55.3 |  |
| 8-9h | 33.9 | 37.3 | 40.7 |  |
| ≥10h | 0.4 | 0.4 | 0.9 |  |
| Phobic anxiety index (%) |  | | | 8.3 |
| CCI: 0-1 | 53.7 | 54.0 | 50.6 |  |
| CCI: 2 | 19.6 | 19.3 | 20.4 |  |
| CCI: 3 | 8.6 | 9.8 | 11.3 |  |
| CCI: 4-5 | 7.9 | 8.8 | 7.1 |  |
| CCI: 6 or higher | 1.9 | 0.8 | 1.1 |  |
| Physical or sexual intimate partner violence (%) | 25.8 | 26.6 | 24.6 | 22.1 |
| Family history of major depression, (%) | 12.3 | 14.1 | 14.6 | 0.0 |
| Medical comorbidity burden, (%) | 0.8 | 0.5 | 0.4 | 0.0 |
| Total activity MET-hours/week |  |  |  |  |
| ^*^ Values are means ± SD or percentages;  ^a^ Northeast: PA, NY, NJ, ME, NH, MA, RI, CT, VT;  ^b^ Midwest: MI, WI, IN, OH, IL, ND, NE, MN, KS, SD, IA, MO;  ^c^ South: MS, KY, AL, TN, SC, WA, NC, DE, MD, FL, VA, GA, WV, AR, TX, OK, LA;  ^d^ West: WY, ID, NM, CO, AZ, MT, UT, NV, AK, WA, OR, HI, CA;  ^e^ Morning: definitely a morning or more of a morning than an evening type;  Evening: definitely an evening or more of an evening than a morning;  Neither: neither morning nor evening type;  ^f^ Crown-Crisp Experiential Index (CCI), score range 0-16. | | | |  |

| **Table S3** Age and age-adjusted characteristics of Nurses` Health Study 2 participants who owned a dog in 2017 and walked the dog in the mornings (N=4,163), by dog walking duration. | | | | |
| --- | --- | --- | --- | --- |
|  | Minutes typically spent outdoors while walking dog in the mornings | | | |
|  | Less than 15 minutes (n=1,370) | 16-30 minutes (n=1,451) | 31+ minutes (n=1,345) | Missing, % |
| Age in years^*^ | 62.3± 4.5 | 62.8± 4.6 | 63.1± 4.6 | 0.0 |
| Race (%) |  |  |  | 0.0 |
| White | 97.9 | 97.8 | 96.8 |  |
| Black | 0.9 | 0.6 | 0.5 |  |
| American indigenous | 0.7 | 0.4 | 0.8 |  |
| Asian | 0.5 | 1.1 | 1.7 |  |
| Hawaiian | 0.1 | 0.2 | 0.2 |  |
| Region of living (%) |  |  |  | 2.5 |
| Northeast^a^ | 36.2 | 30.1 | 23.4 | 0.0 |
| Middlewest^b^ | 24.1 | 26.7 | 25.0 | 0.0 |
| South^c^ | 23.9 | 20.5 | 18.7 | 0.0 |
| West^d^ | 13.3 | 21.4 | 30.6 | 0.0 |
| Married (%) | 76.4 | 77.6 | 82.3 | 0.2 |
| Median family income (in hundred thousands of US Dollar) | 0.9± 0.4 | 0.9± 0.3 | 0.9± 0.3 | 2.0 |
| Retired (%) | 33.8 | 40.5 | 46.7 | 0.0 |
| Number of children (total parity) | 1.9± 1.2 | 1.8± 1.2 | 1.8± 1.2 | 0.0 |
| BMI, (kg/m2) | 26.9± 5.7 | 25.8± 5.1 | 25± 4.6 | 3.9 |
| Alcohol consumption (drinks/d) | 0.5± 0.8 | 0.5± 0.8 | 0.6± 0.8 | 0.4 |
| Smoking status (%) |  |  |  | 0.0 |
| Never or Past smoker | 97.7 | 97.6 | 98.4 |  |
| Current smoker | 2.3 | 2.4 | 1.6 |  |
| Chronotype^e^ (%) |  |  |  |  |
| Morning | 63.1 | 63.4 | 67.7 | 0.0 |
| Evening | 24.2 | 23.9 | 20.8 | 0.0 |
| Neither | 3.6 | 3.9 | 3.8 | 0.0 |
| Hours of sleep (%) |  |  |  | 2.2 |
| <5h | 0.3 | 0.1 | 0.4 | 0.0 |
| 5-7h | 60.8 | 57.7 | 59.8 | 0.0 |
| 8-9h | 35.8 | 38.9 | 37.0 | 0.0 |
| ≥10h | 0.8 | 0.6 | 0.3 | 0.0 |
| Phobic anxiety index (%) |  |  |  | 9.7 |
| CCI: 0-1 | 51.8 | 51.3 | 53.0 | 0.0 |
| CCI: 2 | 18.8 | 21.6 | 18.9 | 0.0 |
| CCI: 3 | 8.7 | 10.0 | 12.0 | 0.0 |
| CCI: 4-5 | 9.1 | 8.3 | 7.1 | 0.0 |
| CCI: 6 or higher | 2.0 | 1.8 | 1.2 | 0.0 |
| Physical or sexual intimate partner violence (%) | 25.8 | 24.5 | 23.3 | 22.1 |
| Family history of major depression, (%) | 13.8 | 14.4 | 13.9 | 0.0 |
| Medical comorbidity burden, (%) | 0.9 | 0.5 | 0.5 | 0.0 |
| Total activity MET-hours/week | 30.5± 30.3 | 36.4± 36.1 | 45.7± 39.8 | 0.0 |
| ^*^ Values are means ± SD or percentages;  ^a^ Northeast: PA, NY, NJ, ME, NH, MA, RI, CT, VT;  ^b^ Midwest: MI, WI, IN, OH, IL, ND, NE, MN, KS, SD, IA, MO;  ^c^ South: MS, KY, AL, TN, SC, WA, NC, DE, MD, FL, VA, GA, WV, AR, TX, OK, LA;  ^d^ West: WY, ID, NM, CO, AZ, MT, UT, NV, AK, WA, OR, HI, CA;  ^e^ Morning: definitely a morning or more of a morning than an evening type;  Evening: definitely an evening or more of an evening than a morning;  Neither: neither morning nor evening type;  ^f^ Crown-Crisp Experiential Index (CCI), score range 0-16. | | | | |

## Results for dog ownership and dog walking related exposures with broader definition of reference groups

| **Table S4** Association of pet ownership and incident depression^(a)^ risk of participants in Nurses` Health Study 2 who were free of depression at baseline and reported on pet ownership. Risk estimates are odds ratios (OR) with 95% confidence intervals (CI).(N=26,169) | | | | |
| --- | --- | --- | --- | --- |
|  | | | **OR (95%CI)** | |
| **Exposure** | **N obs.** | **N case** | **Age adj.** | **MV adj.** |
| Any dog(s) | 9,913 | 182 | 1.09 (0.90, 1.32) | 1.08 (0.89, 1.31) |
| No dog^(b)^ (ref) | 16,256 | 263 | 1.00 | 1.00 |
| Only dog(s) | 7,103 | 125 | 1.02 (0.82, 1.25) | 1.02 (0.83, 1.25) |
| Everything else than ‘only dog(s)’^(c)^(ref) | 19,066 | 320 | 1.00 | 1.00 |
| ^(a)^ Incident depression defined as a self-reported diagnosis of depression or self-reported antidepressants use;  ^(b)^‘No dog(s)’ group contains anyone who declared : ‘no pet’ or ‘cat’ or ‘cat and other pet’ or ‘other pet’;  ^(c)^ Everything else than ‘only dog(s)’ : anyone who declared ‘no pet’ or ‘cat’ or ‘other pet’ or ‘dog and cat’ or ‘dog and other pet’ or ‘dog and cat and other pet’;  N obs.: number of observations used; N case: number of depression cases; Age adj.: age-adjusted model;  MV adj.: model adjusted for age, ethnicity, marital, smoking and retirement status, medical comorbidity, BMI, median family income, region of residence, number of children, alcohol consumption, usual number of hours of sleep, family history of depression, phobic anxiety index, intimate partner's violence and chronotype. | | | | |

| **Table S5:** Association of morning dog walking preferences among dog owners compared to no pet owners and incident depression^(a)^ among the participants of Nurses` Health Study 2. Risk estimates are odds ratios (OR) with 95% confidence intervals (CI).(N=21,975) | | | | | |
| --- | --- | --- | --- | --- | --- |
|  | | | | **OR (95%CI)** | |
| **Exposure** | | **N obs.** | **N cases** | **Age adj.** | **MV adj.** |
| Do you yourself walk that dog in the mornings? | No | 5,672 | 111 | 1.19 (0.94,1.52) | 1.19 (0.94,1.51) |
|  | Yes | 4,163 | 68 | 1.02 (0.77,1.35) | 1.00 (0.76,1.32) |
|  | No pet (ref.) | 12,140 | 190 | 1.00 | 1.00 |
| ^(a)^ Incident depression defined as a self-reported diagnosis of depression or self-reported antidepressants use;  N obs.: number of observations used; N case: number of depression cases; Age adj.: age-adjusted model;  MV adj.: model adjusted for age, ethnicity, marital, smoking and retirement status, medical comorbidity, BMI, median family income, region of residence, number of children, alcohol consumption, usual number of hours of sleep, family history of depression, phobic anxiety index, intimate partner's violence and chronotype. | | | | | |

| **Table S6:** Association of morning dog walking preferences among dog owners compared to no-dog owners and incident depression^(a)^ among the participants of Nurses` Health Study 2. Risk estimates are odds ratios (OR) with 95% confidence intervals (CI).(N=26,088) | | | | | |
| --- | --- | --- | --- | --- | --- |
|  | | | | **OR (95%CI)** | |
| **Exposure** | | **N obs.** | **N cases** | **Age adj.** | **MV adj.** |
| Do you yourself walk that dog in the mornings? | No | 5,672 | 111 | 1.15 (0.92,1.45) | 1.14 (0.91,1.43) |
|  |  |  |  |  |  |
|  | Yes | 4,163 | 68 | 0.99 (0.76,1.29) | 0.98 (0.75,1.27) |
|  |  |  |  |  |  |
|  | No dog (ref.) | 16,253 | 263 | 1.00 | 1.00 |
| ^(a)^ Incident depression defined as a self-reported diagnosis of depression or self-reported antidepressants use;  N obs.: number of observations used; N case: number of depression cases; Age adj.: age-adjusted model;  MV adj.: model adjusted for age, ethnicity, marital, smoking and retirement status, medical comorbidity, BMI, median family income, region of residence, number of children, alcohol consumption, usual number of hours of sleep, family history of depression, phobic anxiety index, intimate partner's violence and chronotype. | | | | | |

| **Table S7:** Association of dog-walking-related behaviors among dog owners and incident depression^(a)^ risk among participants of the Nurses` Health Study 2 stratified by chronotype^(b)^**.** Risk estimates are odds ratios (OR) with 95% confidence intervals (CI). (N=23,738). | | | | | | | | | |
| --- | --- | --- | --- | --- | --- | --- | --- | --- | --- |
|  | | **Morning chronotype** | | | | **Evening chronotype** | | | |
|  | |  |  | **OR (95%CI)** | |  |  | **OR (95%CI)** | |
| **Exposure** | | **N obs.** | **N**  **case** | **Age adj.** | **MV adj.** | **N**  **obs.** | **N**  **case** | **Age adj.** | **MV adj.** |
| Do you yourself walk that dog in the mornings? | No | 3,296 | 54 | 0.98(0.71,1.34) | 0.97 (0.70,1.32) | 1,830 | 50 | 1.62 (1.12,2.31) | 1.61 (1.12,2.30) |
|  | Yes | 2,804 | 41 | 0.90 (0.64,1.27) | 0.87 (0.61,1.21) | 993 | 21 | 1.27 (0.77,2.02) | 1.31 (0.82,2.11) |
|  | No dog (ref) | 10,031 | 161 | 1.00 | 1.00 | 4,784 | 80 | 1.00 | 1.00 |
| ^(a)^ Incident depression defined as a self-reported diagnosis of depression or self-reported antidepressants use;  ^(b)^ Morning: definitely a morning or more of a morning than an evening type;  evening : definitely an evening or more of an evening than a morning;  neither: neither morning nor evening type;  N obs.: number of observations used;  N case: number of depression cases;  Age adj.: age-adjusted model;  MV adj.: model adjusted for age, ethnicity, marital, smoking and retirement status, medical comorbidity, BMI, median family income, region of residence, number of children, alcohol consumption, usual number of hours of sleep, family history of depression, phobic anxiety index and intimate partner's violence. | | | | | | | | | |

## Results for strict definition of depression

| **Table S8**  Association of pet ownership and incident depression^(a)^ risk of participants in Nurses` Health Study 2 who were free of depression at baseline and reported on pet ownership. Risk estimates are odds ratios (OR) with 95% confidence intervals (CI). (N=26,169). | | | | |
| --- | --- | --- | --- | --- |
|  |  |  | **OR(95%CI)** | |
| **Exposure** | **N obs.** | **N case** | **Age adj.** | **MV adj.** |
| **No pet (ref.)** | **12,141** | **28** | **1.00** | **1.00** |
| Any pet(s) | 14,028 | 58 | 1.73 (1.10,2.72) | 1.77(1.15,2.71) |
| Any dog(s) | 9,913 | 37 | 1.56 (0.95,2.55) | 1.60 (1.012.53) |
| Only dog(s) | 7,103 | 22 | 1.32 (0.76,2.31) | 1.35 (0.81,2.24) |
| ^(a)^ Incident depression defined as a self-reported diagnosis of depression **and** self-reported antidepressants use;  N obs.: number of observations used; N case: number of depression cases;  Age adj.: age-adjusted model; MV adj.: model adjusted for age, ethnicity, marital, smoking and retirement status, medical comorbidity, BMI, median family income, region of residence, number of children, alcohol consumption, usual number of hours of sleep, family history of depression, phobic anxiety index, intimate partner's violence and chronotype. | | | | |

| **Table S9**  Association of dog-walking-related behaviors among dog owners and incident depression^(a)^ risk of participants in Nurses` Health Study 2 who were free of depression at baseline and reported on pet ownership. Risk estimates are odds ratios (OR) with 95% confidence intervals (CI).(N=21,975). | | | | | |
| --- | --- | --- | --- | --- | --- |
|  |  |  |  | **OR (95%CI)** | |
| **Exposure** |  | **N obs.** | **N cases** | **Age adj.** | **MV adj.** |
| Do you yourself walk that dog in the mornings? | No | 5,672 | 19 | 1.40 (0.78, 2.52) | 1.48 (0.86, 2.56) |
|  | Yes | 4,163 | 17 | 1.75 (0.97, 3.18) | 1.71 (0.98, 2.98) |
|  | No pet | 12,140 | 28 | 1.00 | 1.00 |
| Minutes typically spent outdoors  while walking dog in the mornings | Less than 15 min. | 1,370 | 4 | 1.00 | 1.00 |
|  | 16-30 min. | 1,451 | 8 | 1.79 (0.58, 5.55) | 1.75 (0.71, 4.33) |
|  | >30 min. | 1,345 | 5 | 1.25 (0.36, 4.28) | 1.29 (0.47, 3.52) |
| When do you typically first take your dog for a walk in the mornings? | Before 7am | 1,585 | 4 | 1.00 | 1.00 |
|  | 7-9am | 1,677 | 4 | 0.96 (0.26, 3.50) | 0.92 (0.33, 2.57) |
|  | After 9am | 898 | 8 | 3.42 (1.10, 10.65) | 3.08 (1.19, 7.94) |
| ^(a)^ Incident depression defined as a self-reported diagnosis of depression **and** self-reported antidepressants use;  N obs.: number of observations used; N case: number of depression cases;  Age adj.: age-adjusted model;  MV adj.: model adjusted for age, ethnicity, marital, smoking and retirement status, medical comorbidity, BMI, median family income, region of residence, number of children, alcohol consumption, usual number of hours of sleep, family history of depression, phobic anxiety index, intimate partner's violence and chronotype. | | | | | |

| **Table S10** Association of pet ownership and incident depression^(a)^ risk of participants in Nurses` Health Study 2 who were free of depression at baseline and reported on pet ownership, stratified by chronotype. Risk estimates are odds ratios (OR) with 95% confidence intervals (CI). (N=23,780). | | | | | | | | |
| --- | --- | --- | --- | --- | --- | --- | --- | --- |
| **Exposure** | **Morning chronotype** | | | | **Evening chronotype** | | | |
|  |  | | **OR (95%CI)** | |  | | **OR (95%CI)** | |
|  | **N obs.** | **N case** | **Age adj.** | **MV adj.** | **N obs.** | **N case** | **Age adj.** | **MV adj.** |
| **No pet (ref.)** | 7,568 | 21 | 1.00 | 1.00 | 3,473 | 7 | 1.00 | 1.00 |
| Any pet(s) | 8,589 | 32 | 1.30 (0.75,2.25) | 1.33 (0.80,2.21) | 4,150 | 21 | 2.36 (1.03,5.42) | 2.28 (1.10,4.73) |
| Any dog(s) | 6,145 | 19 | 1.09 (0.59,2.03) | 1.07 (0.61,1.88) | 2,845 | 14 | 2.29 (0.94,5.57) | 2.30 (1.08,4.87) |
| Only dog(s) | 4,470 | 11 | 0.88 (0.43,1.81) | 0.84 (0.44,1.60) | 1,981 | 9 | 2.19 (0.84,5.72) | 2.14 (0.98,4.69) |
| ^(a)^ Incident depression defined as a self-reported diagnosis of depression **and** self-reported antidepressants use;  ^(b)^ Morning: definitely a morning or more of a morning than an evening type;  evening : definitely an evening or more of an evening than a morning;  neither: neither morning nor evening type;  There were 0 cases of strict depression among ‘no pet owners’ in the ‘neither’ chronotype group. Models did not run.  N obs.: number of observations used; N case: number of depression cases;  Age adj.: age-adjusted model; MV adj.: model adjusted for age, ethnicity, marital, smoking and retirement status, medical comorbidity, BMI, median family income, region of residence, number of children, alcohol consumption, usual number of hours of sleep, family history of depression, phobic anxiety index and intimate partner's violence. | | | | | | | | |
